# Development and evaluation of a Chinese-language newborn feeding hotline: A prospective cohort study

**DOI:** 10.1186/1471-2393-9-3

**Published:** 2009-01-29

**Authors:** Patricia A Janssen, Verity H Livingstone, Bruce Chang, Michael C Klein

**Affiliations:** 1 School of Population and Public Health, University of British Columbia, Child & Family Research Institute, Vancouver, BC, Canada; 2 Department of Family Practice, University of British Columbia, Child & Family Research Institute, Vancouver, BC, Canada; 3 Faculty of Medicine, University of British Columbia, Child & Family Research Institute, Vancouver, BC, Canada

## Abstract

**Background:**

Preference for formula versus breast feeding among women of Chinese descent remains a concern in North America. The goal of this study was to develop an intervention targeting Chinese immigrant mothers to increase their rates of exclusive breastfeeding.

**Methods:**

We convened a focus group of immigrant women of Chinese descent in Vancouver, British Columbia to explore preferences for method of infant feeding. We subsequently surveyed 250 women of Chinese descent to validate focus group findings. Using a participatory approach, our focus group participants reviewed survey findings and developed a priority list for attributes of a community-based intervention to support exclusive breastfeeding in the Chinese community. The authors and focus group participants worked as a team to plan, implement and evaluate a Chinese language newborn feeding information telephone service staffed by registered nurses fluent in Chinese languages.

**Results:**

Participants in the focus group reported a strong preference for formula feeding. Telephone survey results revealed that while pregnant Chinese women understood the benefits of breastfeeding, only 20.8% planned to breastfeed exclusively. Only 15.6% were breastfeeding exclusively at two months postpartum. After implementation of the feeding hotline, 20% of new Chinese mothers in Vancouver indicated that they had used the hotline. Among these women, the rate of exclusive breastfeeding was 44.1%; OR 3.02, (95% CI 1.78–5.09) compared to women in our survey.

**Conclusion:**

Initiation of a language-specific newborn feeding telephone hotline reached a previously underserved population and may have contributed to improved rates of exclusive breastfeeding.

## Background

Preference for formula versus breast feeding among women of Chinese descent, remains a concern in North America. Studies from the US, and Canada have reported breastfeeding rates after hospital discharge as low as 10–30% among Southeast Asian immigrant women compared to 65% in Caucasian women [1,2]. Studies of infant feeding practices from countries of origin for Chinese immigrants suggest that practices related to the hot-cold concept (ying and yang), modesty regarding breastfeeding in public, need to return to employment within a few weeks of giving birth, use of formula as a status symbol, and lack of knowledge about the benefits of breastfeeding contribute to these low rates [3-7]. A recent survey of female university students in Hong Kong indicated that only 63% wanted to breastfeed their future children [8]. Rates of exclusive breastfeeding as low as 26% (Malaysia) [5] and 33% (Taiwan) [9] at six weeks postpartum have been reported in Asian countries. Other studies have indicated that breastfeeding rates among Southeast Asian women decline when they immigrate to North America [10,11].

In Canada, the metropolitan area of Vancouver has the highest proportion of persons of Chinese descent (17%) of all such urban areas in Canada, largely as a result of immigration trends in the past 20 years [12]. Vancouver, BC is home to BC Women's Hospital, the largest maternity centre in Canada. At discharge from BC Women's, the rate of exclusive breastfeeding among new mothers of Chinese descent (Hong Kong, China or Taiwan) has been reported anecdotally to be only 25–30% compared to 70% among women overall.

We engaged in a participatory action study in which we partnered with mothers of Chinese descent and a regional public health board to conceptualize, design, implement and evaluate a novel strategy to support breastfeeding.

## Methods

### Design

Our project employed a participatory action methodology in which the persons who stand to benefit from answering research questions and to whom results will be generalized play an active role in designing the research tool, interpreting results, and designing and evaluating an intervention [13]. Authentic participation in research means sharing in the way research is conceptualized, practiced, and brought to bear on the real world. Action means that groups of people can organize the conditions under which they can learn from their own experiences and make this experience accessible to others.

### Sample

The largest community agency serving Chinese women in Vancouver, SUCCESS (United Chinese Community Enrichment Services Society) [14], recruited a convenience sample of twelve women for a focus group to discuss their preferences and experiences related to infant feeding. All participants had emigrated from Hong Kong during the previous ten years. Seven had given birth in Canada; five in Hong Kong. We conducted the focus group in Cantonese then audiotaped and translated the proceedings. We summarized themes and presented them to participants for confirmation of accuracy and completeness. Participants provided informed, written consent.

### Measures

We undertook a telephone survey to determine whether or not preferences for formula feeding expressed in our focus groups would be validated in a representative sample of Chinese women in Vancouver. In consultation with focus group participants, we designed our survey to ascertain women's beliefs and practices related to infant feeding. Items were generated by the research team, including the authors and focus group members, and by a review of relevant literature. Face validity was evaluated by members of the focus group. The final survey was pilot tested on ten women to assess clarity. No additional revisions were made. The survey consisted of 23 items in the antepartum component and 17 for the postpartum component in addition to socio-demographic items. Each component took approximately twenty minutes to complete. Items related to women's knowledge about advantages or disadvantages about feeding methods, their intended and actual feeding practice and reasons for continuing with their plan or not.

We provided information pamphlets for prospective participants written in English and Chinese script to the offices of 40 obstetricians and family medicine physicians (among whom 30 were also Chinese) providing antenatal care to Chinese women pre-registered to deliver at BC Women's hospital. Women indicating to their physician that they were agreeable to participation received a telephone call from a nurse who was fluent in English, Cantonese, and Mandarin to explain the study and arrange a. time for the telephone interview. Interviews took place during the last trimester (after 30 weeks) of pregnancy with a follow-up call during the second postpartum month. In the follow-up interview we ascertained whether women were able to use their preferred method of infant feeding and if not, what they perceived as facilitators or barriers. Interviews were conducted in the preferred language of the participant and took approximately twenty minutes to complete. Participants in the telephone survey provided informed verbal consent prior to proceeding.

We present survey findings as frequencies. Findings from subgroups within the survey were compared using the Chi square statistic. A p-value of 0.05 or less was denoted as statistically significant. We compared breastfeeding rates among women born in China vs. Hong Kong and among women using the hotline vs. those in our pre-hotline survey using odds ratios. We estimated the odds ratios and corresponding 95% confidence intervals from a logistic regression model.

We reconvened the focus group to review survey findings. After an initial review, we realized that the largest immigrant group among our survey participants was from China. Our community partner, SUCCESS, subsequently recruited three additional women to the focus group who were new mothers and immigrants from China. The new members of the focus group supported the validity of the survey items for women who had immigrated from China. The group then brainstormed ideas based on survey findings for interventions to support breastfeeding among new Chinese mothers. Priority interventions were identified, implemented and evaluated.

Prior to proceeding the study was granted ethical approval by the University of British Columbia Behavioural Ethics Review Board, Certificate B95-0078.

## Results

### Prevalence of breastfeeding

Preference for formula feeding predominated the discussion in the initial focus group and was associated with a belief that breastfeeding was messy, inconvenient, and socially unacceptable. Participants stated that many Chinese women started breastfeeding to comply with their physician's advice, but most found it distasteful and quit within two weeks of giving birth. One participant, who was a bank manager, said "I couldn't believe my eyes" when one of her clients breastfed her baby during a meeting.

For the telephone survey, we identified 566 women who were eligible and willing to participate, according to their maternity care provider. We were able to contact 373 (65.9%) by telephone after a maximum of four attempts, including during weekends and evenings. At least 10% of the phone numbers on the hospital pre-registration lists were incorrect. Among those contacted, 77 were no longer eligible, either because they had already delivered, n = 68 (18.2%) or had a pregnancy loss, n = 9 (2.4%). Of the 296 remaining eligible women, 250 (84%) agreed to participate. At the postnatal survey 196 (78.4% of 250) mothers participated. Twenty-three had returned to Hong Kong or Taiwan. The whereabouts of another 31 were unknown but we were able to ascertain their choice of infant feeding from their hospital record.

Our survey study sample is described in Table 1. Participants were mostly employed outside the home, evenly distributed according to years lived in Canada (< 1 year, 1–4.9, 5.0–9.9, ≥ 10) and roughly half were nulliparous. On average, women were 31 years of age and had 12 years of schooling. Most respondents were born in China (52.7%) and Hong Kong (32.8%), with 6.2% in Taiwan, 4.6% in Canada, and 3.7% in other countries. The majority of participants were interviewed in Chinese languages; 69% in Cantonese and 3.2% in Mandarin. All identified their ethnic origin as Chinese.

**Table 1:** Characteristics of survey participants (n = 250)

	Mean	SD
Age [mean (SD)]	31.2	4.1
Years of Education	12.6	3.6
Spouse's years of education	13.6	4.1
		
	n	%
Employed outside of home n (%)	152	61%
		
Years lived in Canada		
< 1 year	44	17.6
1 – 4.9 years	73	29.2
5 – 9.9 years	69	27.6
10 or more	64	25.6
		
Country born in		
China	127	50.8
Hong Kong	79	31.6
Taiwan	15	6.0
Canada	11	4.4
Other countries	18	7.2
		
Language of interview		
Cantonese	172	68.8
Mandarin	8	3.2
English	70	28.0
		
Parity – primiparous	122	48.8
		
Planning to attend prenatal classes	58	23.2
		
Advice from physician re infant feeding		
Breastfeed	31	34.8
Formula feed	24	27
Unsure of advice	34	38.2
No answer to this question	161	
		
Planned infant feeding method:		
Breastfeeding in combination	126	50.4
Breastfeeding exclusively	52	20.8
Formula exclusively	67	26.8
Unsure	5	2.0

Almost as many women (27%) had received prenatal advice to formula feed from their physicians as to breastfeed (34.8%). (Table 1) Most subjects (83%) thought that breastfeeding would be the best way to feed their infant. (Data not shown). Seventy-one percent planned to start with exclusive breastfeeding. Among those planning to breastfeed, 65.5% planned at some point to combine breastfeeding with formula feeding. Overall, only 29% of the 178 (20.8% overall) planned to breastfeed exclusively until they weaned their baby. During the antenatal period, 52.4% of women planning to breastfeed felt extremely or very confident in their ability to breastfeed, 32.6% felt somewhat confident and 15% felt not very or not at all confident. Chinese vs. Hong Kong immigrants were significantly less likely to plan to breastfeed, odds ratio (OR) 0.43, 95% confidence intervals (CI) (0.20–88). Adjustment for mother's age and years in Canada did not significantly alter these odds ratios.

The trend towards less breastfeeding among Chinese vs. Hong King immigrants continued for exclusive breastfeeding at hospital discharge, OR 0.36, 95% CI (0.20–0.64). Overall, rates of exclusive breastfeeding were 47.2% at hospital discharge and dropped to 15.6% within two months (Table 2). Rates at two months postpartum did not differ by country of origin.

**Table 2:** Breastfeeding experience of survey participants (n = 250)

	n	%
Infant feeding at hospital discharge		
Exclusive breastfeeding	118	47.2
Combined breast and formula	70	28.0
Formula feeding only	62	24.8
		
Infant feeding at two months postpartum		
Exclusive breastfeeding	39	15.6
Combined breast and formula	58	23.2
Formula feeding only	153	61.2
		
Reasons for choosing formula feeding during hospital stay (n = 62)		
Believed they would not have enough milk	30	48.3
Other persons could feed baby	8	12.9
Returning to work	4	6.4
Convenience	2	3.2
No reason given	18	29.0
		
Reasons for introducing formula after breastfeeding initiation(n = 79)		
Believed they did not have enough breast milk	35	44.3
Convenience	6	7.6
Belief that baby would be easier to wean	8	10.1
No reason given	30	38.0
		
Help seeking among women having breastfeeding difficulties (n = 49)		
Physician	12	24.5
Hospital nurse	17	34.7
Lactation consultant	3	6.1
Family member	3	6.1
Hospital drop in clinic	3	6.1
Community health department drop-in clinic	2	4.0
Friend	3	6.1
No one	6	12.2

The predominant reason for choosing formula exclusively or as a supplement while in hospital was a belief that there was not enough breast milk. The majority of women seeking help for breastfeeding did so from hospital nurses, during the first 48–72 hours postpartum.

### Chinese language infant feeding telephone hotline

**Figure 1:**
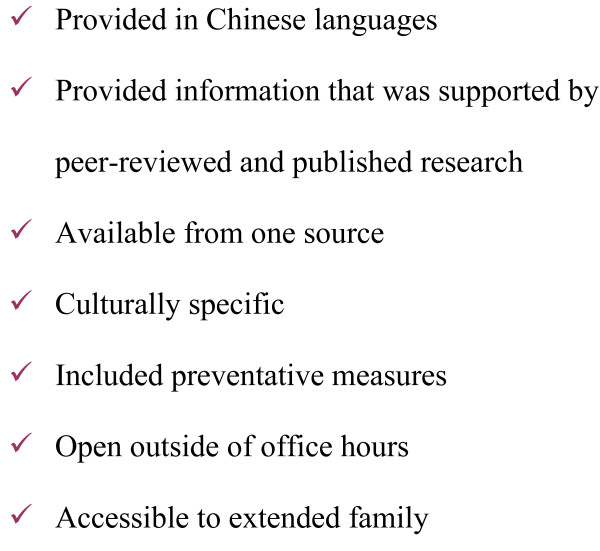
**Attributes of a health promotion program in the Chinese community**.

On review of survey findings, focus group participants developed a list of attributes of an intervention that would promote exclusive breastfeeding (Figure 1). As a priority need, they identified a Chinese language newborn telephone hotline. We then began to plan the implementation of a Chinese language infant feeding telephone hotline in consultation with the regional Vancouver Richmond Health Board (VRHB). The VHRB was already operating an English language hotline. We hired nine registered nurses to staff the hotline. All were experienced community health nurses and six had worked in a perinatal setting. Three were certified lactation consultants. We adapted protocols for responding to and documenting calls from the VRHB hotline. The hotline operated from 1800–2200 hours 7 days per week. Information about the hotline was included both in postpartum information packages distributed at Vancouver hospitals and in packages distributed by public health nurses during routine postnatal home visits to new mothers. The service was advertised through the Chinese language media.

During the first five months of operation, use of the Chinese language hotline increased to 80 calls per month and remained at that level. Women called the hotline with questions related to milk supply, babies spitting up and vomiting, engorgement, sore nipples and many other feeding problems (Table 3). Use of the existing English language hotline did not change. A subsequent survey among women who had previously attended prenatal classes at SUCCESS six months after implementation (unpublished) indicated that 63% of new Chinese mothers were aware of the hotline and 19.4% had used it.

**Table 3:** Characteristics of mothers using hotline

	n	%
Age of mother in years (n = 365 women)		
20–25	20	5.5
26–30	97	26.6
31–35	130	35.6
36–40	86	23.6
41–45	32	8.8
		
Weeks postpartum at time of call (n = 557 calls)		
0–1	109	20.0
2–3	147	27.0
4–5	72	13.2
6–7	38	7.0
8–9	44	8.1
10–19	83	15.2
20–29	36	6.6
30+	16	2.9
Not stated	12	
		
Method of Feeding at time of call		
Breastfeeding exclusively:	246	44.1
Formula feeding exclusively:	121	21.8
Combination feeding	190	34.1

Among 365 individual women using the hotline, the rate of exclusive breastfeeding overall was 44.1%.(Table 3) This compares to 15.6% among women in our telephone survey at two months postpartum, odds ratio 3.02, 95% CI (1.78–5.09). Among 229 women who stated they were breastfeeding, 36 (11.4%) were providing breast milk via a bottle; they were not putting the baby to the breast. In spite of the fact that the service was specifically called a feeding hotline, there were many other areas of inquiry (Table 4).

**Table 4:** Mother's concerns precipitating a call to the hotline

	n	%
Breastfeeding	226	40.4
Inadequate milk supply	42	
Spitting up/vomiting	31	
Engorgement	28	
General information	22	
Supplementation	17	
Mother on medication	15	
Sore nipples	13	
Weaning	11	
Baby's appetite decreasing	10	
Hiccoughs	10	
Baby not satisfied	7	
Mastitis	6	
Baby sleepy at breast	5	
Use of vitamins	5	
Slow weight gain	2	
Latching problems	2	
		
Infant Health/Illness	102	18.3
Infant Behaviour	49	8.8
Infant Elimination	126	22.6
Infant Sleep	12	2.2
Formula Feeding	89	16.0
Feeding Solids	44	7.9
Infant Crying	27	4.8
Infant Care	61	11.0
Immunization	16	2.9
Maternal Health	56	10.1
Medication	14	2.5
Community Resources	4	0.7
Other	29	5.3

Interventions were undertaken by the hotline nurses in response to 490 (88%) of calls: advice provided by the hotline nurses 413 (84.3%); referral to a physician 63 (12.8%); referral to community health nurses 7 (1.3%); breastfeeding drop-in clinics 4 (0.8%); and a nutritionist 1 (0.3%). Two clients were directed to go immediately to a hospital emergency room. Six months after the conclusion of our study, the VRHB reopened the hotline as part of their services to the community on a permanent basis.

## Discussion

The initial findings from our focus group, that culturally specific beliefs play an influential role in women's preferences for infant feeding, were not supported by our survey of 250 women. The primary reason for introducing formula reported in our survey was not unique to Chinese culture but common to many new mothers; that is, a concern that they would not have enough milk. Studies focusing on Asian women have reported similar concerns among new mothers [5,15]. Concern about insufficient milk supply, therefore can not explain entirely the difference in breastfeeding rates observed in our population prior to the study, unless women of Chinese descent do not receive the necessary teaching and support to address this misinformation. Of concern was the small proportion of women who had received encouragement to plan to breastfeed. This barrier to breastfeeding has been reported in other recent studies as well [6].

Our findings of low rates or exclusive breastfeeding at hospital discharge (47%) and at two months postpartum (15.6%) are not different than other studies addressing this population. Recent studies from Hong Kong report breastfeeding initiation rates as high as 60% [6] but only about 22% at two months postpartum [16]. A study of immigrant Chinese mothers in Australia reported breastfeeding rates of 34% at three months and recommended ethno-specific services to support breastfeeding for the first six months postpartum [17].

The Chinese Language Newborn Feeding Hotline enjoyed a rapid uptake. Requests for information were for general, as opposed to culturally specific information. Unchanged rates of usage of the English language hotline indicated that the hotline was addressing a previously underserved population. Since most calls were received within the first month of life, the hotline was supplementing care given by family physicians, pediatricians, and community health nurses.

Rates of exclusive breastfeeding were threefold greater in women using the newborn hotline compared to women at two months postpartum in our pre-hotline survey. Our findings may be biased in that only women seeking help with feeding accessed the hotline and women determined to breastfeed may preferentially use this service. The telephone service was never described, however, as a breastfeeding hotline, and women received assistance to formula feed if they so requested. Another potential limitation to our study is that our pre-intervention survey, unlike the Newborn Hotline, was hospital-based, rather than population-based. However, approximately 80% of the births in the City of Vancouver take place at BC Women's Hospital.

A report on multicultural perspectives on infant feeding in Canada described variation among Chinese women according to country of origin[12]. In this report, women who had come from Hong Kong were more likely to be informed abut the benefits of breastfeeding, and were more likely to initiate and continue breast feeding compared to women from Mainland China and Taiwan. Our survey supports these findings. It is possible that women from Hong Kong may have been more likely to access the Newborn Hotline, and if so, our findings may not be generalizable to women of all Chinese immigrant groups. Initiatives to target women from China specifically may be warranted in future studies.

It is worth noting that a variety of calls about issues not directly related to infant feeding indicate an unmet need among this population for assistance with parenting issues other than breastfeeding.

## Conclusion

We utilized a participatory action approach in a community-based study to address disproportionately low rates of exclusive breastfeeding among immigrant Chinese women. Our focus groups and a survey of Chinese women before and after giving birth directed the development of a Chinese-language telephone information service for newborn feeding. Women using the hotline had a threefold greater odds of breastfeeding exclusively. While we cannot attribute these increased rates in a causal fashion to the hotline due to the cross-sectional nature of our surveys, our results should encourage the implementation and evaluation of telephone-accessed language-specific services in other public health settings serving new mothers.

## Abbreviations

CI: Confidence Intervals; OR: Odds Ratios; SUCCESS: United Chinese Community Enrichment Services Society; VRHB: Vancouver Richmond Health Board.

## Competing interests

The authors declare that they have no competing interests.

## Authors' contributions

PJ contributed to the design of the study, participated in focus groups, undertook data analysis, organized the newborn hotline service, and wrote the first draft of the manuscript. VL contributed to the design of the study, with a major contribution to the design of surveys, interpretation of focus group findings, and development of the newborn hotline. BC originally outlined the need for the study and conceptualized the design. He assisted in interpretation of study findings. MK participated in developing the study design, facilitated data collection, participated in interpretation of findings. All authors contributed to the writing of the manuscript and have read and approved the final version.

## Pre-publication history

The pre-publication history for this paper can be accessed here:


